# Central Arterial Hemodynamic Effects of Dark Chocolate Ingestion in Young Healthy People: A Randomized and Controlled Trial

**DOI:** 10.1155/2014/945951

**Published:** 2014-05-27

**Authors:** T. Pereira, J. Maldonado, M. Laranjeiro, R. Coutinho, E. Cardoso, I. Andrade, J. Conde

**Affiliations:** ^1^Departamento de Cardiopneumologia, ESTESC, Instituto Politécnico de Coimbra, Rua 5 de Outubro, SM Bispo, Apartado 7006, 3046-854 Coimbra, Portugal; ^2^Instituto de Investigacao e Formacao Cardiovascular, Quinta da Portela, Rua Princesa Cindazunda, Lote 9.3, Loja 94, 3030-503 Coimbra, Portugal

## Abstract

*Introduction.* The aim of this study was to assess the vascular benefits of dark chocolate in healthy and young individuals. * Methods*. A randomized and controlled trial was carried out involving 60 healthy volunteers, randomized into two groups: control group (CG; *n* = 30) and intervention group (IG; *n* = 30). The IG ingested a daily dosage of 10 g of dark chocolate (>75% cocoa) for a month. Blood pressure (BP), flow-mediated dilation (FMD), arterial stiffness index (ASI), aortic pulse wave velocity (PWV), and pulse wave analysis (PWA) were assessed at baseline and one week after the one-month intervention period. * Results*. Arterial function improved after intervention in the IG, with PWV decreasing from 6.13 ± 0.41 m/s to 5.83 ± 0.53 m/s (*P* = 0.02), with no significant differences observed in the CG. A significant decrease in ASI (0.16 ± 0.01 to 0.13 ± 0.01; *P* < 0.001) and AiX (−15.88 ± 10.75 to −22.57 ± 11.16; *P* = 0.07) was also depicted for the IG. Endothelial function improved in the IG, with the FMD increasing 9.31% after the 1-month intervention (*P* < 0.001), with no significant variation in the CG. * Conclusion*. The daily ingestion of 10 g dark chocolate (>75% cocoa) during a month significantly improves vascular function in young and healthy individuals.

## 1. Introduction

Cardiovascular diseases represent an important worldwide public health problem, being the main cause of mortality and morbidity [1]. Recent studies have shown that the severity of endothelial dysfunction relates to the risk for an initial or recurrent cardiovascular event [2].

The endothelium acts to maintain vascular homeostasis through multiple complex interactions with cells in the vessel wall and lumen. The endothelium regulates vascular tone (balancing the production of vasodilators and vasoconstrictors), controls blood fluidity and coagulation (producing factors that regulate platelet activity, the cloth cascade, and the fibrinolytic system), and produces cytokines and adhesion molecules (regulate the inflammatory process) [2]. There are multiple risk factors, such as smoking, ageing, hypercholesterolemia, hypertension, hyperglycemia, and a family history of premature atherosclerotic disease, all of them associated with a loss of endothelium-dependent vasodilation.

Nutrition has long been thought to have a profound effect on endothelial function [3]. Indeed, epidemiologic evidence supports the idea that diets rich in fruits and vegetables promote health and attenuate the onset of various diseases, including cardiovascular disease [4].

In the context of cardiovascular disease, flavan-3-ol (flavanols) has received considerable attention. Cocoa and chocolate products have a much higher flavan-3-ol concentration and total antioxidant capacity per weight than other flavonoid-containing beverages such as red wine and green or black tea [4, 5]. Flavonoid-rich chocolate markedly improves coronary vasodilation, indicative of an increased bioavailability of NO (ability to improve endothelial function), and decreases platelet reactivity (ability to decrease blood clotting) [1, 4, 6, 7]. Therefore a diet rich in flavonoids might have the potential to improve cardiovascular health [7–9].

In the last few years, several randomized studies have been published assessing the effects of food containing cocoa, specifically chocolate, in intermediary endpoints of cardiovascular disease. The consumption of dark chocolate or cocoa drinks rich in flavonoids has been shown to improve endothelial function and reduce blood pressure and LDL oxidation [5–7, 10–13]. In terms of vascular physiology, short term consumption of flavonoid-rich dark chocolate reduces the wave reflection, improving endothelial function and cardiovascular risk in healthy adults [5]. This suggests a possible cardioprotective effect by flavonoid-rich chocolate, even in healthy persons [11].

Considering the increasing body of epidemiologic evidence stating the potential benefits of flavonoids, we design a randomized and controlled trial aiming at ascertaining whether a daily ingestion of a small amount of cocoa-rich chocolate (>70%) improves the vascular function in young and healthy individuals. Dark chocolate was used because it is formulated with a higher percentage of cocoa bean liquor; therefore it contains greater amounts of flavonoids as compared with other alternatives [5]. In this study, we used aortic pulse wave velocity (PWV), central pulse wave analysis (PWA), and flow-mediated dilation (FMD) as surrogates of the potential vascular benefits of dark chocolate in young and healthy people [14].

## 2. Methods

### 2.1. Population

Between November 2012 and February 2013, 60 clinically healthy individuals of Portuguese nationality (10 men and 20 women), all undergraduate students at the Superior Polytechnic Institute of Coimbra, under the age of 25 years, were enrolled and were randomly allocated to one of two groups: control group (CG) and intervention group (IG). Randomization was done in accordance with a computer-generated randomization list, done with the SAS program, version 9.2 (SAS Institute Inc., Cary, NC, USA). The subjects were allocated to each group following the randomization list. The control group consisted in 30 subjects aged between 18 and 24 years (mean age: 20.67 ± 1.64 years). The intervention group consisted in 30 subjects aged between 18 and 23 years (mean age: 19.90 ± 1.70 years). The gender distribution was similar in both groups. All subjects were told to maintain their normal daily routine and were all free of chronic medication.

The aims of the trial were explained to all participants and their informed consent was obtained.

### 2.2. Study Design

An experimental, randomized, and controlled trial was done to study the vascular effects of dark chocolate in young people. A sample of 60 young and healthy individuals was randomized into two groups (CG and IG) with similar clinical features, and subjects were followed, with two clinical evaluations: first evaluation: baseline; second evaluation: one week after the end of the intervention period, a month, during which the IG ingested a 10 g/day dosage of dark chocolate with over 75% cocoa. The CG had no concomitant intervention.

Clinical evaluations included carotid-femoral PWV, arterial PWA, brachial FMD, blood pressure (BP), heart rate (HR), and clinical observation. At each consultation, the subjects' weight and height were measured and body mass index (BMI) was calculated in kg/m^2^. BP was measured in a supine position and after a 10-minute resting period, by an experienced operator and using a clinically validated (class A) sphygmomanometer (Colson MAM BP 3AA1-2; Colson, Paris) [15]. The mean of three BP measurements was used in the analysis. In the first evaluation (baseline), all individuals answered a short clinical questionnaire that focused on personal and family background, smoking habits, alcohol and medication consumption, and vegetables intake.

The 10 g/day dosage of dark chocolate with over 75% cocoa was given by an element of the research team on a daily basis (between 16 h and 18 h p.m.), and the type of chocolate was the same for all volunteers. All subjects were undergraduate students at the Superior Polytechnic Institute of Coimbra and had their regular meals at the university canteen from Monday to Friday; therefore the overall nutritional patterns were similar for all participants.

### 2.3. Pulse Wave Velocity, Pulse Wave Analysis, and Flow-Mediated Dilation

Carotid-femoral PWV was determined using the Complior Analyze (Alam Medical, France) in accordance with a previously described technique [16]. Briefly, PWV was based on the distance/time ratio (meters/second) with the pulse wave measured simultaneously in the right carotid and right femoral arteries, the distance used being that between the sites where the pressure waves were recorded.

Carotid PWA was also performed with the Complior Analyze (Alam Medical, France) device, simultaneously with the PWV measurement. For PWA, the Complior's carotid sensor was positioned with its specific hand-free holder over the right carotid artery, at the point of maximum signal amplitude, stability, and detail. A 15-second window was used to acquire the pulse waveforms. Central blood pressures and augmentation indexes were then extracted for analysis [11].

PWA was further analysed with the Vasograph device (DigitalMed, Hungary), which uses a novel technique of near infrared photoplethysmography over the digital arteries and provides reliable measures of arterial stiffness and vascular tone [17]. This was performed with the patient in a supine position; the probe was positioned in the right pointing finger, and a 1 minute acquisition window was used. The arterial stiffness index (ASI) was then extracted for analysis.

A Vivid 3 echograph (General Electric Company, USA) was used for the determination of brachial FMD, with a linear vascular transducer of 7 MHz, according to a previously described method [6, 9]. The FMD was measured with the patient in a supine position, after resting in a tranquil environment. Some restrictions were made, such as the ingestion of caffeine and/or smoking in the 2 hours prior to the assessment. The right brachial artery was scanned longitudinally and identified with color flow Doppler imaging, 2 to 5 cm above the antecubital fossa. The diameter of the brachial artery was measured by calculating the distance between the inner proximal and distal intimal layers (D1) at telediastole. Ischemia was provoked through pneumatic compression of the radial and cubital arteries during 5 minutes. The cuff was positioned 2 to 3 cm below the antecubital fossa and was insufflated 30 mmHg above the previously measured systolic blood pressure. About 60 seconds after the cessation of the ischemic period, the brachial artery diameter was measured again (D2). The FMD was obtained according to the formula: (D2 − D1)/D1 × 100. Endothelium-independent vasodilation was not performed.

Measurements were blindly performed by the same operator, and the quality of the recordings was evaluated by two independent and blind observers, with considerable experience regarding the method.

### 2.4. Statistical Analysis

All statistical analysis was performed with SPSS for Windows, version 19.0. The distribution of the variables was tested for normality using the Kolmogorov-Smirnoff test and for homogeneity of variance by Levene's test. Simple descriptive statistics were used to characterize the sample and the distribution of variables.

The comparisons between groups were made through the *χ* ^2^ test, with Fisher correction when appropriate, for categorical variables, or Student's* t*-test for independent samples, for normally distributed quantitative variables.

For* within-subject* comparisons, we used Student's* t*-test for pairwise samples, or repeated measures ANOVA, carried through the several considered measures, as best suited. For the repeated measures ANOVA, the Greenhouse-Geisser correction for the degrees of freedom was adopted whenever* sphericity* violation was verified. All multiple comparisons meant to localize the significant effects of a factor were based on the Bonferroni correction.

The criterion of statistical significance used was *P* ≤ 0.05 for a confidence interval of 95%.

## 3. Results

The study population consisted of 60 healthy individuals, randomized into two groups, with similar proportion of males and females. The groups did not differ significantly in the several anthropometric and clinical characteristics taken into account. Baseline group characteristics are shown in Table 1. The adherence rate of dark chocolate intake was above 99%.

Table 2 summarizes the data obtained for each group over the two moments of clinical evaluation. No significant differences were observed between groups for all the considered variables over the baseline evaluation. Also, there were no significant variations over time regarding BMI, HR, and brachial BP in both groups, although a consistent trend for BP reduction was identified in the IG (cf. Table 2). A statistically significant decrease in the PWV was seen in the IG (6.13 ± 0.41 m/s at baseline and 5.83 ± 0.53 m/s after intervention; *P* = 0.02), but not in the CG (6.28 ± 0.95 m/s at baseline and 6.40 ± 1.13 m/s after intervention; *P* = 0.22). A statistically significant decrease in the ASI was also found in the IG (0.16 ± 0.01 at baseline and 0.13 ± 0.01 after intervention; *P* < 0.01), with no significant variation over the CG. Similar finding was also documented for the augmentation index (AiX).

As for the FMD variation, there were no significant differences between the baseline values in both groups (*P* = 0.535). However, the FMD improved considerably after the one-month intervention period in the IG, with no significant variations in the CG. The FMD increased from 13.91 ± 4.71% to 23.22 ± 7.64% in the IG (*P* < 0.001), with no relevant variations in the CG (*P* = ns).

**Figure 1:**
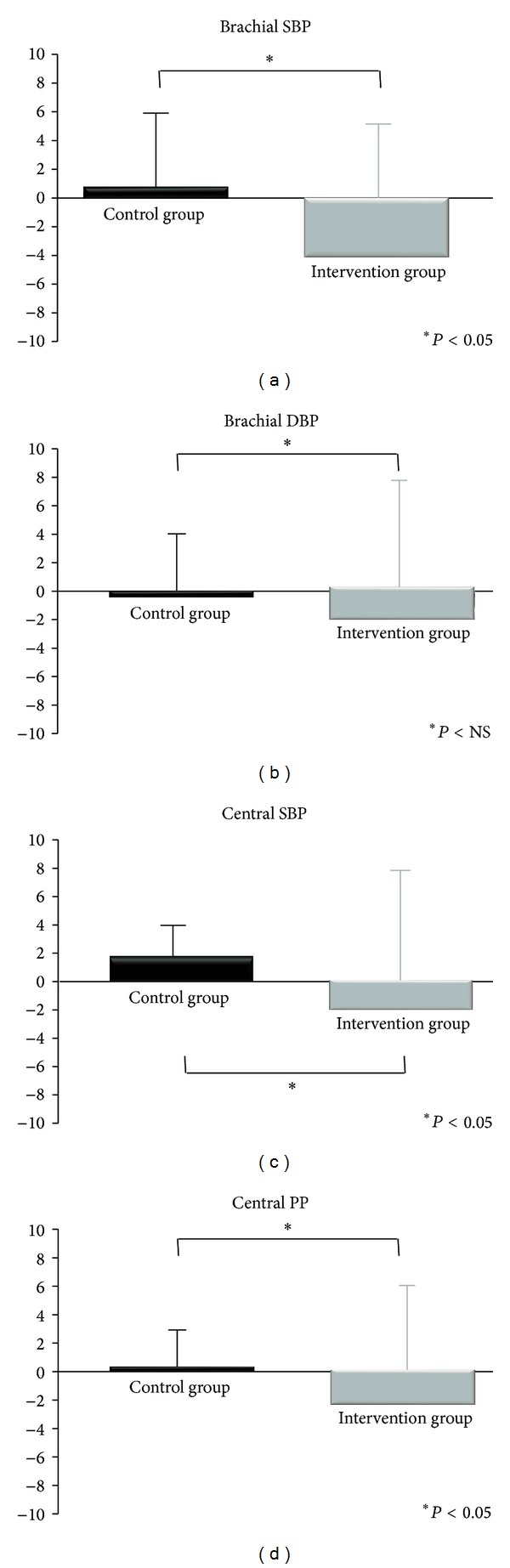
Differences in brachial and central blood pressure after 30 days of chocolate consumption. SBP—systolic blood pressure; DBP—diastolic blood pressure; PP—pulse pressure.

**Figure 2:**
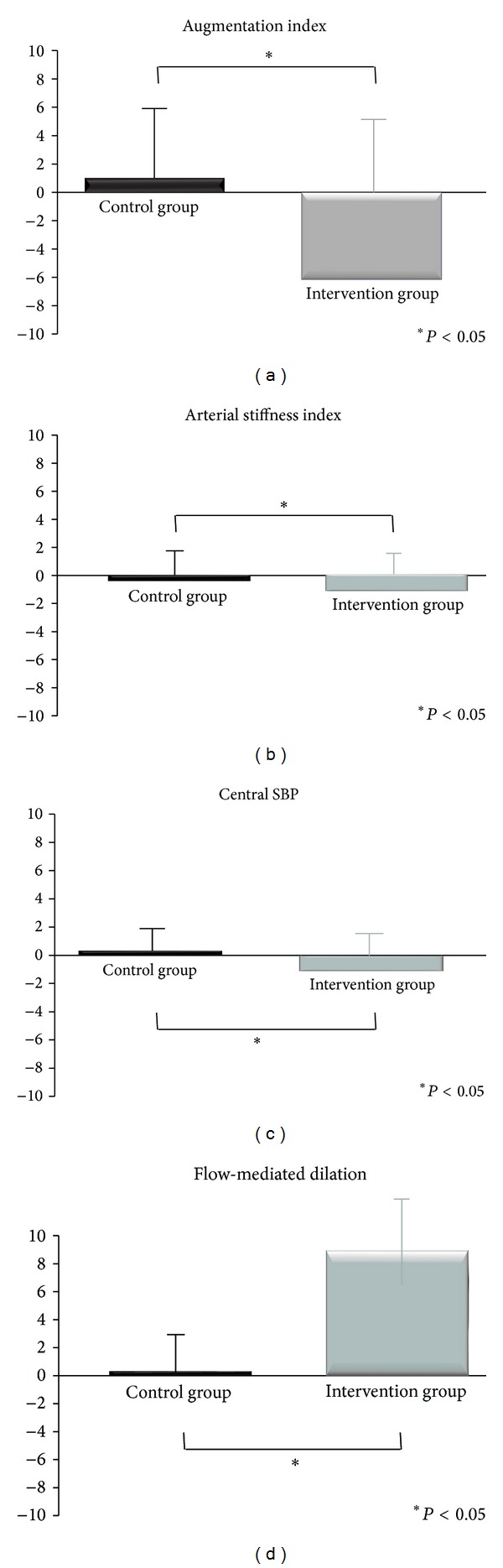
Differences in central hemodynamic parameters after 30 days of chocolate consumption.

Considering the mean within-group individual differences pooled for each group, all variables demonstrated a reduction after the one-month intervention in the IG, with statistically significant effects for ASI and PWV (*P* < 0.001 and *P* = 0.010, resp.). A reduction was also found for brachial and central BP (Figures 1 and 2).

As a summary, a significant improvement was depicted in different parameters of vascular function in the IG after the 10 g/day dark chocolate intake, particularly considering the PWV, the AiX, the ASI, and the FMD. Contrariwise, no significant variations were found in the CG.

## 4. Discussion

Endothelial dysfunction constitutes the initial step of atherosclerosis [18], depending largely on the decreased bioavailability of endothelial nitric oxide (NO), a key determinant for vascular vasomotricity. The endothelial impairment plays a critical role, not only in atherosclerotic processes but also in arteriosclerosis, leading to a continuous loss of arterial compliance. Therefore, arterial stiffness has been considered an increasingly important biomarker in the evaluation of cardiovascular risk [14]. In fact, as the aorta stiffens, the PWV increases, and the reflected pressure wave reaches the heart earlier, causing an important augmentation of the central systolic blood pressure (cSBP) and thus increasing the cardiac afterload.

Recent studies suggest that the consumption of flavonoid-rich food, like dark chocolate, may improve the endothelial function [5, 7, 13]. Murphy et al. [3] showed that the consumption of dark chocolate by healthy people, during 28 days, increases plasma concentrations of catechins and epicatechins and modifies platelet function (reducing platelet aggregation, mean platelet volume, and platelet degranulation and increasing plasma concentration of ascorbic acid). Engler et al. [6] also described the augmentation in the concentration of plasmatic epicatechin with the consumption of dark chocolate during 2 weeks. This increase was associated with improved endothelial function. On the other hand, several studies revealed no changes in biomarkers of antioxidant and oxidative stress (such as LDL oxidation, ORAC—oxygen radical absorbance capacity, TAC—total antioxidant capacity, and MDA—circulating malondialdehyde) after the consumption of dark chocolate [5, 6].

In the study by Faridi et al. [7], the acute consumption of dark chocolate or cocoa powder significantly lowered the brachial BP, and the magnitude of these changes was greater with the consumption of sugar-free cocoa. In contrast, no statistically significant changes in BP were found in our study after the intervention, although a consistent trend for BP reduction was seen in the IG but not in the CG. In fact, when the two groups were compared in terms of individual differences for the two moments of evaluation, the brachial and central BP significantly decreased in the IG as compared with the CG (cf.  Figure 1).

In the study performed by Vlachopoulos et al. [5], the AiX, and tendentiously, the PWV, decreased, reaching its maximum after 180 minutes of consumption of dark chocolate (74% cocoa). A similar finding was found in our study, with a reduction of the AiX and PWV and, furthermore, the ASI, after the consumption of dark chocolate for a month. The decrease in AiX reveals a reduction in the ratio of the reflected wave to the incident wave. This could possibly translate the PWV and ASI reduction, indicating greater arterial distensibility/relaxation, so that slower wave velocity results in later reflected waves to the heart. Wave reflections are in fact important determinants of coronary blood flow and cardiovascular performance [5]. Enhanced wave reflections have been identified as independent markers of cardiovascular morbidity and mortality. In fact, with arterial stiffening we can observe two adverse effects on the central circulation and the interaction between the left ventricle and aorta: one is the direct effect caused by decreased aortic compliance and the other is an indirect effect which causes increased pressure in the aorta through early reflected waves. The consequent increased afterload promotes left ventricular hypertrophy and increases left ventricle myocardial oxygen consumption [19].

Our study clearly shows that, even in healthy young people with normal vascular function, arterial benefits associated with the daily consumption of dark chocolate can be found. The probable mechanism underlying the improvement of PWV, ASI, and AiX observed after cocoa consumption may in fact be the parietal relaxation of the large arteries (primarily affecting the aortic PWV and the ASI), as well as a dilation of small and medium sized peripheral arteries and arterioles (primarily affecting the reflected waves and also the ASI), due to an increased concentration of plasma epicatechin, which increases endothelium-derived vasodilators and also increases the concentration of plasma procyanidins, which in turn leads to the rise in NO production and bioavailability [6, 20]. The concomitant finding of improved FMD strongly suggests endothelium-dependent vascular relaxation as the motive for the vasomotor benefit found, translating into lower PWV and ASI and lower AiX and for a trend in BP reduction.

Our study may have important clinical implications, since a growing body of evidence focuses on the dietary flavonoids potential to decrease cardiovascular risk. Our findings provide a mechanism according to which chocolate may exert a protective effect on the cardiovascular system. Our study indicates a promising dietary option to promote better arterial function and to reduce the cardiovascular risk associated with its deterioration. In fact, other studies have also reported benefits of flavanol-containing cocoa in clinical patient populations. For example, Balzer et al. [21] studied its benefits in the vascular function of medicated diabetic patients and observed that the fasting plasma levels of flavanols metabolites increased significantly, and also endothelial function was shown to improve. In contrast, another study failed to demonstrate benefits of a daily consumption of cocoa beverages and bars, neither in the endothelial function nor in systemic arterial compliance (SAC), despite the demonstration of increased plasma epicatechin concentration [8]. These diverging results indicate that the benefits of dark chocolate are better seen in early or intermediate stages of the atherosclerotic process, failing to benefit patients with overt and irreversible atherosclerotic disease. In this sense, we can suggest flavanol-containing cocoa as a promising and powerful option for cardiovascular primary prevention.

Cocoa-rich chocolate contains several substances in addition to flavanols [5], which theoretically could have influenced the results. Thus, the observed changes may be the result of the beneficial effects of flavonoids, although we cannot exclude the participation of other substances.

The present study shows that the daily consumption of 10 gr of dark chocolate (>70% cocoa) for a month globally improves the vascular function, promoting better arterial distensibility indexes, delaying the reflected waves, and improving the overall ventricular-vascular interaction features. Although chocolate consumption demonstrated a protective effect on the cardiovascular system, further studies are needed to assess the long-term effects of cocoa on cardiovascular risk reduction and vascular function.

Since this study refers to healthy young subjects, further studies are needed to confirm our findings in other populations.

## Figures and Tables

**Table 1:** Baseline study cohort characteristics.

	Total (*n* = 60)	Control group (*n* = 30)	Intervention group (*n* = 30)	*P*
Age, years (mean ± SD)	20.23 ± 2.22	20.67 ± 2.67	19.8 ± 1.70	0.29
BMI, Kg/m^2^ (mean ± SD)	22.92 ± 3.66	23.66 ± 3.41	22.17 ± 3.87	0.28
Gender (*n*/%)				
Female	40/66.7	20/66.7	20/66.7	1.00
Male	20/33.3	10/33.3	10/33.3	
Smoke (*n*/%)				
Yes	6/10.0	4/13.3	2/6.7	0.50
No	54/90.0	26/86.7	28/93.3	
Family history (*n*/%)				
Yes	40/66.7	22/73.3	18/60.0	0.35
No	20/33.3	8/26.7	12/40.0	
Total cholesterol, mg/dL (mean ± SD)	161.38 ± 21.65	161.33 ± 21.66	161.43 ± 21.62	0.93
Triglycerides, mg/dL (mean ± SD)	105.99 ± 16.64	106.14 ± 16.61	105.92 ± 16.66	0.84
Creatinine, mg/dL (mean ± SD)	0.65 ± 0.08	0.65 ± 0.07	0.65 ± 0.08	0.92
Glycemia, mg/dL (mean ± SD)	82.65 ± 8.45	82.22 ± 9.03	83.24 ± 7.15	0.91

BMI: body mass index; HR: heart rate; DBP: diastolic blood pressure; SBP: systolic blood pressure; SD: standard deviation; bpm: beats per minute.

**Table 2:** Variation of the clinical parameters per group and per assessment moment.

	Control group (*n* = 30)			Intervention group (*n* = 30)		
	Baseline	30 days	*P*	Baseline	30 days	*P*
BMI, Kg/m^2^	23.55 ± 3.51	23.63 ± 3.49	0.22	22.18 ± 3.87	22.15 ± 3.92	0.61
HR, bpm	75.00 ± 15.19	74.15 ± 19.07	0.90	72.20 ± 10.30	72.20 ± 9.78	1.00
bSBP, mmHg	120.00 ± 12.56	121.57 ± 12.00	0.62	120.33 ± 7.67	116.67 ± 7.48	0.10
bDBP, mmHg	68.21 ± 7.50	68.07 ± 6.84	0.92	67.67 ± 6.78	66.00 ± 6.60	0.42
cSBP, mmHg	109.92 ± 13.66	111.46 ± 12.25	0.90	110.6 ± 8.49	109.00 ± 8.37	0.53
cPP, mmHg	43.00 ± 10.95	43.00 ± 9.99	1.00	44.92 ± 10.52	42.33 ± 10.18	0.19
AiX, %	−17.54 ± 13.78	−16.15 ± 15.72	0.68	−15.88 ± 10.75	−22.57 ± 11.16	0.07
ASI,	0.15 ± 0.02	0.14 ± 0.07	0.10	0.16 ± 0.01	0.13 ± 0.01	0.00
PWV, m/s	6.28 ± 0.95	6.30 ± 1.13	0.52	6.13 ± 0.41	5.83 ± 0.53	0.02
FMD, %	12.85 ± 4.51	13.23 ± 5.76%	0.70	13.91 ± 4.71%	23.22 ± 7.64%	0.00

AiX: augmentation index; ASI: distensibility index; BMI: body mass index; bDBP: brachial diastolic blood pressure; bSBP: brachial systolic blood pressure; cPP: central pulse pressure; cSBP: central systolic blood pressure; HR: heart rate; PWV: pulse wave velocity; FMD: flow-mediated dilation.

## Abbreviations

AiX: Augmentation index
AP: Augmentation pressure
ASI: Distensibility index
BMI: Body mass index
BP: Blood pressure
CG: Control group
DBP: Diastolic blood pressure
HR: Heart rate
IG: Intervention group
LDL: Low density lipoprotein
MDA: Circulating malondialdehyde
NO: Nitric oxide
ORAC: Oxygen radical absorbance capacity
PWV: Pulse wave velocity
SAC: Systemic arterial compliance
SBP: Systolic blood pressure
TAC: Total antioxidant capacity.
